# Impact of visual presentation of atherosclerotic carotid plaque on cardiovascular risk profile using mHealth technologies

**DOI:** 10.1038/s41746-024-01423-y

**Published:** 2025-01-22

**Authors:** Greta Ullrich, Alexander Bäuerle, Lisa Maria Jahre, Katrin Paldán, Jana Rosemeyer, Chiara Kalaitzidis, Christos Rammos, Martin Teufel, Tienush Rassaf, Julia Lortz

**Affiliations:** 1 Department of Cardiology and Vascular Medicine, West-German Heart and Vascular Center Essen, University of Duisburg-Essen, Hufelandstr. 55, 45147 Essen, Germany; 2 Clinic for Psychosomatic Medicine and Psychotherapy, LVR-University Hospital Essen, Virchowstr. 175, 45147 Essen, Germany; 3 Center for Translational Neuro- and Behavioral Sciences (C-TNBS), University of Duisburg-Essen, Essen, Germany; 4 UCT Research, Vorarlberg University of Applied Science, Hochschulstraße 1, 6850 Dornbirn, Austria

**Keywords:** Risk factors, Cardiovascular diseases, Vascular diseases, Atherosclerosis

## Abstract

This randomized, controlled trial evaluated the impact of plaque visualization combined with daily tasks on cardiovascular risk profile and included 240 participants with coronary arterial disease. The intervention group received the *PreventiPlaque* app during the 12-month study period in addition to standard care. The app contained daily tasks that promoted lifestyle modifications and adherence to prescribed medication. It included ultrasound images of participants´ individual carotid plaque, which were updated regularly. The impact of plaque visualization and personalized app usage was evaluated, using a change in the SCORE2 as a primary endpoint. In the intervention group, the SCORE2 was significantly lower after the study period (*t*(120) = 6.43, *p*_adj_ < 0.001, *d*_RM_ = 0.58). This demonstrates the efficacy of the *PreventiPlaque* app in supporting lifestyle modifications and medication adherence. These findings suggest that personalized mHealth interventions in combination with visual risk communication are valuable tools in secondary prevention. *Trial Registration*: The study was registered at ClinicalTrials.gov under the identifier NCT05096637 on 27 October 2021 and was approved by the local ethics committee of the University of Duisburg-Essen (20-9157-BO).

## Introduction

The prevalence of atherosclerotic cardiovascular disease remains at an all-time high and it is still the leading cause of death worldwide^1^. Coronary artery disease and ischemic heart disease alone lead to 16% of total deaths per year worldwide and have been the diseases with the most rapidly growing share in overall death rates worldwide during the last 20 years^1^. Apart from the high mortality rates, cardiovascular disease is also linked to many comorbidities, impacts the health-related quality of life and leads to a severe mental burden^2,3^. Within the last years, diagnostic as well as therapeutic interventions for acute, life-threatening cardiovascular events have been improving with the progress in the medical field, which leads to increasing survival rates^4^. This can cause more chronic courses of disease and makes optimal secondary prevention as the most important cornerstone in chronic disease management indispensable. According to current guideline recommendations, secondary prevention should include the treatment of modifiable cardiovascular risk factors such as arterial hypertension, hypercholesterolemia, and diabetes mellitus. Moreover, smoking cessation and a healthy diet, as well as regular physical activity and stress reduction should be encouraged^5^. Although implementing these lifestyle changes could prevent disease progression and lead to stable chronic disease, they continue to be insufficiently conducted and especially long-term adherence remains low. Reasons for this are a shortage of medical resources and consultation time frames to sufficiently educate patients as well as a lack of adherence to behavioral changes^6,7^. Digital health interventions have been emerging during the last years with an increase of digitalization in the medical field and are gaining importance and various scopes. Meeting the before-mentioned current barriers, they represent a powerful tool to further educate and enable patients to take a more active role in chronic disease management. This way, they have been shown to improve patients‘ adherence to prescribed medication^8^. To be most effective, a level of personalization is required since feelings of patient involvement and self-efficacy are promoted by personalized digital health interventions, leading to better chances at long-term behavioral lifestyle changes^9,10^. Therefore, the *PreventiPlaque* app was designed following a patient-centered approach. Since the visualization of atherosclerotic plaque has been shown to improve preventive measures^11^, we included personalized pictorial presentation of the participants‘ own atherosclerotic carotid plaque as well as low-threshold daily tasks within the app to promote active patient involvement and, therefore, a feeling of self-efficacy. The aim of this trial was to evaluate whether visual plaque presentation, conveyed via a personalized mHealth technology, in combination with daily tasks to promote positive lifestyle changes and medication adherence, can impact the overall cardiovascular risk profile (Table 1).

**Table 1:** Sociodemographic and medical characteristics at baseline

	Total sample	Intervention group	Control group
	*N* (%)	*N* (%)	*N* (%)
Gender			
Male	141 (58.8)	64 (52.9)	77 (64.7)
Female	96 (40.0)	56 (46.3)	40 (33.6)
NA	3 (1.2)	1 (0.8)	2 (1.7)
Marital status			
Single	26 (10.8)	14 (11.6)	12 (10.1)
In a relationship	26 (10.8)	15 (12.4)	11 (9.2)
Married	141 (58.8)	71 (58.7)	70 (58.8)
Separated/divorced	27 (11.2)	17 (14.0)	10 (8.4)
Widowed	15 (6.2)	4 (3.3)	11 (9.2)
NA	5 (2.1)	0 (0.0)	5 (4.2)
Educational level			
No degree	6 (2.5)	5 (4.1)	1 (0.8)
Lower secondary education	69 (28.7)	36 (29.8)	33 (27.7)
Higher secondary education	70 (29.2)	38 (31.4)	32 (26.9)
Higher education entrance qualification	52 (21.7)	27 (22.3)	25 (21.0)
University education	34 (14.2)	12 (9.9)	22 (18.5)
Other	3 (1.2)	2 (1.7)	1 (0.8)
NA	6 (2.5)	1 (0.8)	5 (4.2)
Occupational status			
Unemployed	19 (7.9)	7 (5.8)	12 (10.1)
Sick leave	9 (3.8)	6 (5.0)	3 (2.5)
Part-time employed	22 (9.2)	10 (8.3)	12 (10.1)
Full-time employed	53 (22.1)	31 (25.6)	22 (18.5)
Retired	120 (50.0)	61 (50.4)	59 (49.6)
NA	17 (7.1)	6 (5.0)	11 (9.2)
Place of residence (population size)			
Large city (>100,000 residents)	190 (79.2)	92 (76.0)	98 (82.4)
Medium-sized city (>20,000 residents)	29 (12.1)	20 (16.5)	9 (7.6)
Small town (>5000 residents)	12 (5.0)	7 (5.8)	5 (4.2)
Rural area (<5000 residents)	4 (1.7)	2 (1.7)	2 (1.7)
NA	5 (2.1)	0 (0.0)	5 (4.2)
BMI (kg/m^2^)	28.55 (5.87)	29.26 (6.67)	27.82 (4.84)
Smoking			
Currently	60 (25.0)	34 (28.1)	26 (21.8)
In the past	76 (31.7)	37 (30.6)	39 (32.8)
Coronary artery bypass graft (CABG)	22 (9.2)	17 (14.0)	5 (4.2)
Percutaneous coronary intervention (PCI)	100 (41.7)	50 (41.3)	50 (42.0)
Peripheral artery disease (PAD)	88 (36.7)	43 (35.5)	45 (37.8)
Peripheral bypass	10 (4.2)	6 (5.0)	4 (3.4)
Percutaneous transluminal angioplasty	50 (20.8)	27 (22.3)	23 (19.3)
Chronic vein insufficiency	14 (5.8)	8 (6.6)	6 (5.0)
Amputation	2 (0.8)	0 (0.0)	2 (1.7)
Deep vein thrombosis	13 (5.4)	9 (7.4)	4 (3.4)
Pulmonary embolism	9 (3.8)	4 (3.3)	5 (4.2)
Acute coronary syndrome	35 (14.6)	21 (17.4)	14 (11.8)
Angina pectoris	25 (10.4)	15 (12.4)	10 (8.4)
Congestive heart failure	63 (26.2)	29 (24.0)	34 (28.6)
Valvular disease	62 (25.8)	26 (21.5)	36 (30.3)
Intervention	7 (2.9)	1 (0.8)	6 (5.0)
Stroke	14 (5.8)	6 (5.0)	8 (6.7)
Transient ischemic attack	1 (0.4)	1 (0.8)	0 (0.0)
Type 2 diabetes	41 (17.1)	26 (21.5)	15 (12.6)
Diabetes mellitus type 1 or 2	41 (17.1)	23 (19.0)	18 (15.1)
Arterial hypertension	193 (80.4)	95 (78.5)	98 (82.4)
Pulmonary hypertension	4 (1.7)	2 (1.7)	2 (1.7)
Hyperlipidemia	191 (79.6)	92 (76.0)	99 (83.2)
Hyperuricemia	8 (3.3)	3 (2.5)	5 (4.2)
Medication			
Blood pressure	179 (74.6)	96 (79.3)	83 (69.7)
Cholesterol	170 (70.8)	94 (77.7)	76 (63.9)
Aspirin (acetylsalicylic acid)	123 (51.2)	65 (53.7)	58 (48.7)
Mental illness	39 (16.2)	21 (17.4)	18 (15.1)
Dropout	41 (17.1)	17 (14.0)	24 (20.2)
Total	*N* = 240	*N* = 121	*N* = 119

*NA* data not available, *BMI* body mass index.

## Results

### Study sample

In total, 240 patients with atherosclerotic cardiovascular disease participated in the study. Of the sample, 121 were allocated to the intervention group, and the control group consisted of 119 participants. Overall, 17.1% (41) of participants dropped out of the study. The number of dropouts did not significantly differ between the intervention group (14.0%, 17) and the control group (20.2%, 24; *χ*^2^(1) = 1.2, *p* = 0.277).

The mean age was 61.9 years (SD = 9.0; intervention group: *M* = 61.7, SD = 8.8; control group: *M* = 62.1, SD = 9.3), with ages ranging between 34 and 84 years. Of the total sample, 58.8% (141) of the participants were male.

### Primary outcome: Change in the overall cardiovascular risk profile (SCORE2)

Primary and secondary outcomes in both groups at all five measure points are reported in Table 2. There were no significant differences between the intervention and control group in baseline values for primary and secondary outcomes (*p* > 0.05), except for diastolic blood pressure, which was significantly higher in the intervention group, *t*(238) = −2.12, *p* = 0.035, and quality of life, which was significantly higher in the control group, *t*(238) = 2.09, *p* = 0.038.

**Table 2:** Primary and secondary outcomes for all measurepoints by group

		Baseline (t0)	3-month follow-up (t1)	6-month follow-up (t2)	9-month follow-up (t3)	12-month follow-up (t4)
Outcome	Group	*M* (SD)	*M* (SD)	M (SD)	M (SD)	M (SD)
Cardiovascular risk profile (SCORE2)	Intervention control	9.91 (6.86) 8.77 (5.22)	9.06 (5.70) 9.15 (4.85)	8.80 (5.37) 8.72 (4.90)	7.75 (4.54) 8.52 (4.89)	7.51 (4.43) 8.65 (4.95)
LDL-C	Intervention control	106.79 (67.76) 95.88 (41.05)	96.90 (49.85) 95.74 (46.59)	89.98 (45.80) 94.89 (39.86)	88.31 (48.22) 96.47 (40.83)	75.23 (39.92) 86.14 (37.85)
HbA1c	Intervention control	5.98 (0.77) 5.93 (0.57)	6.08 (0.98) 5.99 (0.58)	6.03 (0.82) 6.04 (0.67)	5.98 (0.72) 6.14 (0.82)	5.98 (0.77) 6.02 (0.73)
Systolic blood pressure	Intervention control	149.00 (23.99) 142.63 (22.46)	138.51 (18.96) 144.98 (19.77)	138.33 (19.09) 139.98 (16.03)	134.38 (16.40) 143.23 (16.79)	131.68 (15.64) 141.25 (16.75)
Physical activity	Intervention control	220.17 (300.45) 245.74 (381.36)	254.89 (432.15) 249.50 (377.75)	269.26 (269.32) 235.76 (232.03)	378.76 (626.23) 281.26 (280.22)	291.40 (595.00) 169.98 (175.15)
Medication adherence	Intervention control	4.32 (1.51) 4.19 (1.39)	3.82 (1.09) 4.18 (1.31)	3.83 (1.24) 4.03 (1.27)	3.74 (1.07) 3.90 (1.14)	3.74 (1.00) 3.82 (1.01)
Quality of life (EuroQoL)	Intervention control	56.69 (25.55) 63.04 (21.30)	61.72 (22.72) 64.83 (20.29)	62.29 (23.69) 64.76 (21.03)	63.28 (22.82) 63.11 (22.20)	63.59 (22.54) 58.11 (22.54)

Intervention group: *N* = 121; control group: *N* = 119. SCORE2: Systematic Coronary Risk Evaluation; Physical activity: Minutes per week; Medication adherence: Lower scores indicate higher adherence; EuroQoL: European Quality of Life 5 Dimensions 5 Level Version questionnaire.

**Fig. 1:**
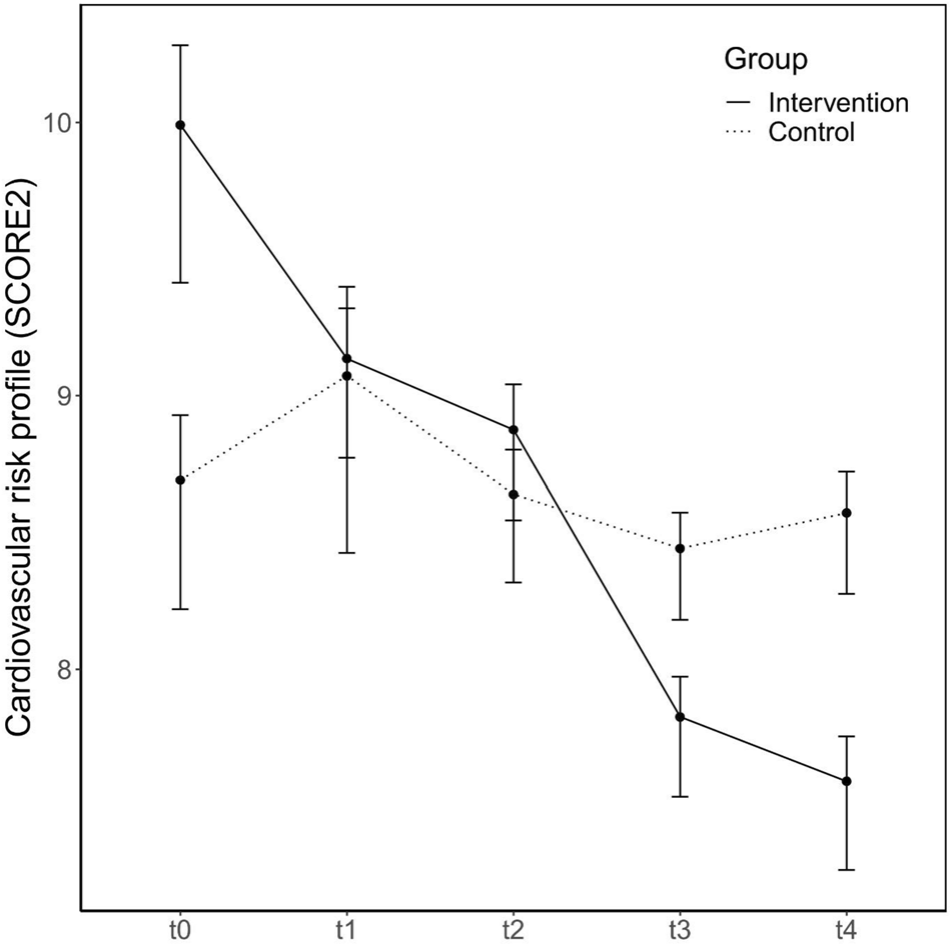
Change in the overall cardiovascular risk profile (SCORE2) from baseline to 12-month follow-up. The graph illustrates the time-course changes in the SCORE2 (Systematic COronary Risk Evaluation), a measure of cardiovascular risk, for participants in the intervention group (solid line, *n* = 121) and the control group (dotted line, *n* = 119). From baseline to the final follow-up, the intervention group showed a marked and progressive decline in SCORE2 values, indicating a significant reduction in cardiovascular risk. In contrast, the control group exhibited minimal changes, with SCORE2 levels slightly fluctuating over time but remaining near their baseline levels. The divergence between the two groups became most pronounced after 6 months (*t*2), with the intervention group achieving the lowest risk profile after 12 months (*t*4). Error bars represent 95% confidence intervals for adjusted within-subject standard errors. *t*0: baseline examination, *t*1: 3 months follow-up, *t*2: 6 months follow-up, *t*3: 9 months follow-up, *t*4: 12 months follow-up.

**Fig. 2:**
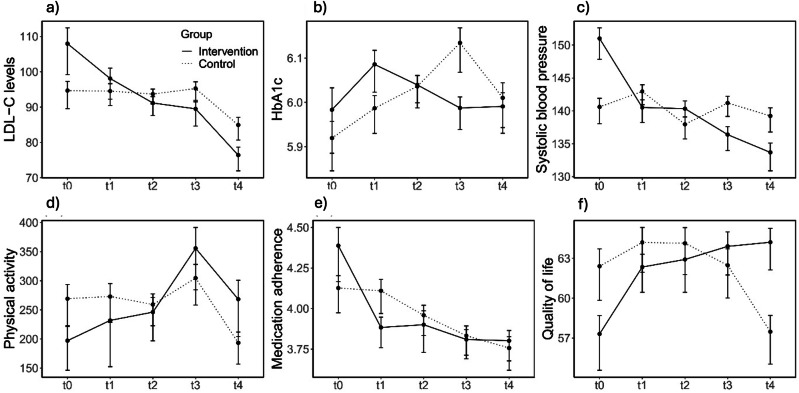
Changes in lifestyle alterations from baseline to 12-month follow-up. Changes in lifestyle alterations from baseline (*t*0) to 12-month follow-up (*t*4) in intervention (*n* = 121) and control groups (*n* = 119). **a** LDL-C levels (mg/dl): The intervention group experienced a progressive and significant reduction in LDL-C levels over time compared to the control group, with marked improvements evident by *t*4. **b** HbA1c levels (%): Glycemic control, as indicated by HbA1c, fluctuated over time, with the intervention group showing improved stability after an initial rise after 3 months (*t*1), whereas the control group displayed more variable trends. **c** Systolic blood pressure (mmHg): A consistent decrease in systolic blood pressure was observed in the intervention group, achieving significant reductions by *t*4, while the control group showed no substantial change. **d** Physical activity (minutes per week): The intervention group exhibited a notable increase in physical activity after 6 months (*t*2), followed by a decline but maintained higher levels than baseline. The control group showed minimal variability. **e** Medication adherence (lower scores indicate higher adherence): Both groups showed a gradual decline in medication adherence over time, though the intervention group demonstrated slightly better maintenance of adherence, particularly at earlier time points. **f** Quality of life: The intervention group experienced a gradual improvement in quality-of-life scores, surpassing the control group by *t*4. Variability was observed within both groups at intermediate time points. This figure illustrates the longitudinal effects of the intervention on cardiovascular risk factors and lifestyle-related outcomes, demonstrating greater improvements in the intervention group across most domains. Statistical significance (*p*-values) and effect sizes were not shown but are described in the text. Error bars represent 95% confidence intervals for adjusted within-subject standard errors. t0: baseline examination, *t*1: 3 months follow-up, *t*2: 6 months follow-up, *t*3: 9 months follow-up, *t*4: 12 months follow-up.

Figures 1 and 2*(title) visualize the results of the ANOVAs*.

A two-way mixed ANOVA revealed a significant main effect on SCORE2 for time and a significant interaction effect for group × time. A robust two-way mixed ANOVA confirmed the significant main effect for time (*p* < 0.001), and the significant interaction effect for group × time (*p* < 0.001). Posthoc tests revealed a significant effect of time on SCORE2 for the intervention group (*p*_adj_ < 0.001), but not the control group, *p*_adj_ > 0.05. Posthoc pairwise comparisons between time points showed that SCORE2 was significantly higher at *t*0 compared to *t*2 (*p*_adj_ = 0.017) compared to *t*3 (*p*_adj_ < 0.001) and compared to *t*4 (*p*_adj_ < 0.001) for the intervention group only.

### Secondary outcomes: Change in lifestyle alterations

#### LDL-C

A two-way mixed ANOVA revealed a significant main effect on LDL-C levels for time (*p* < 0.001) and a significant interaction effect for group × time (*p* < 0.001). A robust two-way mixed ANOVA confirmed the significant main effect for time (*p* < 0.001) and the significant interaction effect for group × time (*p* = 0.001). Posthoc tests revealed a significant effect of time on LDL-C levels for the intervention group (*p*_adj_ < 0.001) and the control group (*p*_adj_ = 0.008).

#### HbA1c

A two-way mixed ANOVA revealed a significant main effect on HbA1c for time (*p* = 0.018) and a significant interaction effect for group × time (*p* = 0.007). A robust two-way mixed ANOVA confirmed the significant main effect for time (*p* = 0.017). The interaction effect for group × time was not confirmed (*p* = 0.109). Posthoc tests revealed a significant effect of time on HbA1c for the control group (*p*_adj_ = 0.001), but not the intervention group (*p*_adj_ > 0.05). Posthoc pairwise comparisons between time points showed that HbA1c was significantly higher at t3 compared to t0 (*p*_adj_ = 0.003), compared to t1 (*p*_adj_ = 0.037) and compared to t4 (*p*_adj_ = 0.016) for the control group.

#### Systolic blood pressure

A two-way mixed ANOVA revealed a significant main effect on systolic blood pressure for time (*p* < 0.001), a significant main effect for group (*p* < 0.001), and a significant interaction effect for group × time (*p* < 0.001). A robust two-way mixed ANOVA confirmed the significant main effect for time (*p* < 0.001), the main effect for group (*p* = 0.038), and the significant interaction effect for group × time (*p* < 0.001). Posthoc tests revealed a significant effect of group on systolic blood pressure, with a significant difference between groups at *t*3 (p_adj_ < 0.001) and at *t*4 (*p*_adj_ < 0.001). Further, posthoc tests revealed a significant effect of time on systolic blood pressure for the intervention group (*p*_adj_ < 0.001), but not the control group (*p*_adj_ > 0.05).

#### Physical activity

A two-way mixed ANOVA revealed a significant main effect on physical activity for time (*p* = 0.010). A robust two-way mixed ANOVA confirmed the significant main effect for time (*p* < 0.001) and revealed a significant interaction effect for group × time (*p* = 0.03). However, posthoc tests indicated no significant effect of time on physical activity for both groups (*p*_adj_ > 0.05).

#### Medication adherence

A two-way mixed ANOVA revealed a significant main effect on medication adherence for time (*p* < 0.001), and a significant interaction effect for group × time (*p* = 0.042). A robust two-way mixed ANOVA confirmed the significant main effect for time (*p* < 0.001), and the significant interaction effect for group × time (*p* = 0.038). Posthoc tests revealed a significant effect of time on medication adherence for the intervention group (*p*_adj_ < 0.001) and the control group (*p*_adj_ = 0.002).

#### Quality of life

A two-way mixed ANOVA revealed a significant main effect on quality of life for time (*p* = 0.011) and a significant interaction effect for group × time (*p* < 0.001). A robust two-way mixed ANOVA confirmed the significant main effect for time (*p* = 0.007), and the significant interaction effect for group × time (*p* < 0.001). Posthoc tests revealed a significant effect of time on quality of life for the intervention group (*p*_adj_ = 0.002), and the control group (*p*_adj_ = 0.006).

## Discussion

Implementing healthy lifestyle modifications is a crucial aspect of secondary prevention and management of chronic cardiovascular conditions^12^. To achieve stable disease control, it is necessary to reduce and optimally manage risk factors, such as obesity, arterial hypertension, hypercholesterinemia, and nicotine consumption over the course of decades^12^. This requires long-term behavioral changes and adherence to prescribed medication. However, disease management often fails due to a lack of treatment adherence^7^.

Studies have demonstrated the potential benefit of visually presenting personalized pictorial information that represents the current disease status or progression^11,13,14^. Personalized health information can enhance patient involvement in chronic disease management by fostering a sense of self-efficacy, which is essential for long-term behavior changes^10,15^. This study is the first to combine visual plaque presentation with personalized mHealth technology, making health information accessible to participants at any time and further improving patient participation (Table 3).

**Table 3:** Posthoc pairwise comparison between timepoints

	Intervention group			Control group		
	Posthoc pairwise comparison between timepoints (*t*0; *t*2)	Posthoc pairwise comparison between timepoints (*t*0;*t*3)	Posthoc pairwise comparison between timepoints (*t*0; *t*4)	Posthoc pairwise comparison between timepoints (*t*4; *t*1)	Posthoc pairwise comparison between timepoints (*t*4; *t*2)	Posthoc pairwise comparison between timepoints (*t*4; *t*3)
LDL-C	*t*(120) = 3.52 *p*_adj_ = 0.006 *d*_RM_ = 0.29	*t*(120) = 3.30 *p*_adj_ = 0.013 *d*_RM_ = 0.27	*t*(120) = 6.27 *p*_adj_ < 0.001 *d*_RM_ = 0.51	*t*(118) = 2.97 *p*_adj_ = 0.036 *d*_RM_ = 0.31	*t*(118) = 3.22 *p*_adj_ = 0.002 *d*_RM_ = 0.30	*t*(118) = 4.08 *p*_adj_ = 0.017 *d*_RM_ = 0.39
Systolic blood pressure	*t*(120) = 5.27 *p*_adj_ < 0.001 *d*_RM_ = 0.44	*t*(120) = 6.22 *p*_adj_ < 0.001 *d*_RM_ = 0.50	*t*(120) = 7.20 *p*_adj_ < 0.001 *d*_RM_ = 0.56	*t*(120) = 3.38 *p*_adj_ = 0.01 *d*_RM_ = 0.27	*t*(120) = 3.62 *p*_adj_ = 0.004 *d*_RM_ = 0.30	–
Medication adherence	*t*(120) = 3.17 *p*_adj_ = 0.02 *d*_RM_ = 0.27	*t*(120) = 4.20 *p*_adj_ < 0.001 *d*_RM_ = 0.34	*t*(120) = 4.19 *p*_adj_ < 0.001 *d*_RM_ = 0.32	*t*(118) = 3.06 *p*_adj_ = 0.028 *d*_RM_ = 0.33	–	–
Quality of life	*t*(120) = 2.98 *p*_adj_ = 0.035 *d*_RM_ = 0.26	*t*(120) = 3.23, *p*_adj_ = 0.016 *d*_RM_ = 0.28	*t*(120) = 3.64 *p*_adj_ = 0.004 *d*_RM_ = 0.32	*t*(118) = 3.58 *p*_adj_ = 0.005 d_RM_ = 0.31	*t*(118) = 3.48 *p*_adj_ = 0.007 *d*_RM_ = 0.31	*t*(118) = 4.94 *p*_adj_ < 0.001 *d*_RM_ = 0.45

After using the *PreventiPlaque* app for 12 months, we observed a statistically significant improvement in the overall cardiovascular risk profile, quantified by the SCORE2, compared to the control group. This indicates a significant risk reduction for cardiovascular events in the upcoming years for patients with access to the *PreventiPlaque* app. Additionally, secondary endpoints such as systolic blood pressure and LDL-C levels showed positive impacts following the intervention period. However, there was no statistically significant improvement in the examined endpoints physical activity and HbA1c levels. Therefore, using a personalized mHealth technology seems to be a suited tool to include visual information in risk communication and, therefore improve secondary prevention for patients with cardiovascular disease. In the future, this concept could be implemented and tested in primary prevention as well.

Improving long-term adherence to physical activity has been a persistent challenge in secondary preventive measures for cardiovascular conditions. Studies indicate that adherence to recommended activity levels typically decreases over time following an acute event such as myocardial infarction or stroke^16^. Even among the average middle-aged German population, levels of physical activity are highly insufficient^17^. This could explain why we could not observe a significant increase in self-reported physical activity levels within the study population. Additionally, the *PreventiPlaque* app was not specifically designed to increase physical activity, lacking features such as a pedometer or wearables to monitor exercise behavior. Including such features could potentially enhance adherence to physical activity recommendations in future versions of the application.

Another secondary outcome was the change in HbA1c levels during the 12-month follow-up period. We observed no significant difference in this regard between the intervention and control groups. This may be attributed to the fact that the *PreventiPlaque* app was not specifically tailored for diabetic patients and lacked features specifically designed for individuals with type 2 diabetes. Although one of the daily tasks involved maintaining a healthy diet, the measures may have been insufficient, and the nutritional recommendations were too broad. Moreover, antidiabetic medication was not adjusted by study personnel during the study period. Generally, mHealth applications specifically tailored to patients with type 2 diabetes have shown great potential in improving glycemic control and lowering HbA1c levels^18^.

In conclusion, to further enhance the *PreventiPlaque* app, it would be necessary to personalize the daily tasks more effectively. This could include providing more detailed nutritional information and additional materials specifically tailored to patients with type 2 diabetes. Moreover, incorporating wearable devices or a pedometer to better track physical activity could be beneficial.

On the contrary, the effect on controlling cardiovascular risk factors, such as arterial hypertension and hypercholesterinemia seemed to be adequate. Medication adherence to lipid-lowering therapy after an acute cardiac event is often described as insufficient^19^. We assume that the visual presentation of individual atherosclerotic plaque within the *PreventiPlaque* app met the need for adequate personalization of health information, leading to an improvement in treatment adherence.

The last secondary endpoint examined was a possible change in health-related quality of life. After the follow-up period, the intervention group showed a significant improvement in their overall health-related quality of life compared to the control group. This improvement could be attributed to an increased feeling of self-efficacy stemming from app-usage and taking an active role in disease management. An association between an increased feeling of self-efficacy and improvement in health-related quality of life has been demonstrated in the past^20,21^.

This trial is limited by the fact that owning a smartphone suitable for app usage was a precondition to participate. This inherently reduces the extent to which these results can be generalized, as patients without smartphones were excluded. Moreover, it should be noted that medication adherence and physical activity were self-reported parameters, which are prone to potential bias.

In conclusion, the *PreventiPlaque* app, which uniquely integrates visual feedback of atherosclerotic plaques with personalized health tasks, significantly improved cardiovascular risk profiles, blood pressure, cholesterol levels, medication adherence, and overall quality of life in the intervention group after one year.

## Methods

### Study design and patient collective

The *PreventiPlaque* trial was a two-armed, parallel randomized, single-center clinical trial which was conducted at the University Clinic of Essen, West German Heart and Vascular Center, Department of Cardiology and Vascular Medicine. Inpatients as well as outpatients were recruited and after giving informed consent to participate and comply with the study protocol and having undergone the baseline examinations, participants were randomly assigned into the intervention or control group using a one-to-one allocation ratio. The study interval lasted for twelve months and patients were asked to attend to a total of four follow-up visits 3 (*t*1), 6 (*t*2), 9 (*t*3) and 12 months (*t*4) after the baseline examination (*t*0). Both groups received the best medical treatment according to current guidelines, including a 5-min standardized verbal briefing on secondary prevention during the baseline visit^5^. Afterwards, the participants who were randomly assigned to the intervention group received access to the *PreventiPlaque* app for the duration of the study period as well as a short introduction on how to use the digital intervention. Within the *PreventiPlaque* app, the intervention group had permanent access to their most recent ultrasound images picturing carotid artery plaque. We recently published a study protocol with more in-depth information about the design of the *PreventiPlaque* app^22^. The control group received the same medical treatment as the intervention group, however without access to the *PreventiPlaque* app during the study period. The control group was only briefly presented with ultrasound images of the carotid artery during the ultrasound examination that took place during the follow-up visits, as it is daily clinical practice in our clinic.

Patients of adult age (≥18 years) with atherosclerotic cardiovascular disease were eligible for participation in this trial. This included patients with documented acute coronary syndrome as well as patients with stable angina, coronary revascularization, proven peripheral artery disease, or a history of stroke^23^. In addition, ultrasound evidence of atherosclerotic plaque in one or both carotid arteries was necessary. The presence of carotid plaque was defined as focal thickening of the vessel wall measuring 50% more than the regular vessel wall or regional outlined carotid intima-media thickness (CIMT) bulging more than 1.5 mm into the lumen of the vessel^24^. Regarding the inclusion criteria, possession of a smartphone suitable for app usage and the ability to use a smartphone and mobile technology had to be provided. Finally, the motivation and commitment to comply with the study protocol, as well as written, informed consent were required. Patients with congestive heart failure with New York Heart Association (NYHA) III–IV symptoms, severe valve disease, insufficient knowledge of the German language, or unwillingness to use the app or submit to diagnostic procedures and attend follow-up visits were excluded from the trial.

### Study aims, research questions, and outcomes

In this randomized controlled clinical trial, we aimed to analyze the effect of a mHealth intervention, which provided the study participants with a pictorial visual presentation of their atherosclerotic carotid plaque. We tested the hypothesis that this plaque presentation and implementation of a personalized mHealth technology would improve patients‘ adherence to secondary preventive therapy and would lead to positive lifestyle changes. We tested whether the regular usage of the mHealth technology would lead to an improvement in cardiovascular risk profiles within the follow-up period as a result.

This study aimed to answer the following research questions:

1. Does visual atherosclerotic plaque presentation using a personalized mHealth intervention impact the participant's cardiovascular risk profile in comparison to a control group?
2. Does using the *PreventiPlaque* app have a measurable effect on controlling cardiovascular risk factors (hyperlipidemia, arterial hypertension, diabetes mellitus) and lead to positive lifestyle changes (increase in physical activity, medication adherence) and an improvement of the quality of life?

The primary outcome of this study was a change in the overall cardiovascular risk profile during the 12-month follow-up period. We defined the primary outcome as a change in the SCORE2 (Systematic Coronary Risk Evaluation) risk calculator^25^ and therefore compared the estimated risk at the time of the baseline visit as well as the follow-up visits (Research Question 1). The SCORE2 contains the individual's age in years, sex, nicotine consumption, HDL-, LDL-C- and total-cholesterol levels, as well as the systolic blood pressure. For the secondary outcome measures we aimed to assess lifestyle alterations made within the intervention period. Therefore, we analyzed changes in physical activity and medication adherence. Patients were asked to self-assess their medication adherence as well as their level of physical activity and duration during the week. To assess medication adherence, we used a self-developed questionnaire that consisted of three items (“How often do you forget to take your medication?”; “How often do you change the prescribed dosage yourself, e.g. because it makes you feel better?”; “How often do you consciously decide not to take your medication, e.g. because you feel worse after taking it?”). We assessed the intake of the regular intake of aspirin, antihypertensives and cholesterol-reducing medication. Moreover, changes in clinical parameters such as systolic blood pressure, body weight, HbA1c, LDL-C-, HDL- and total-cholesterol levels were assessed. Finally, we assessed changes in the participant's quality of life using the European Quality of Life 5 Dimensions 5 Level Version questionnaire (Research Question 2)^26^.

Participants were randomly allocated into a control group and intervention group using the R package ‘randomizer’^27^. Randomization was conducted within blocks to ensure an equal number of smokers and non-smokers in the intervention and control group. The *PreventiPlaque* app was only available in the intervention group; therefore blinding of study participants was not possible. However, the study personnel were blinded during baseline and follow-up visits. Before the randomization, patients were instructed not to talk about the *PreventiPlaque* app during each in-person follow-up visit if they were allocated to the intervention group to enable adequate blinding. The baseline, as well as all four follow-up visits took place at the Department of Cardiology and Vascular Medicine. At the first medical visit, patients willing to participate in the *PreventiPlaque* trial received a short medical briefing and attended baseline examinations after they had provided written consent and were screened regarding the inclusion criteria. The standardized medical briefing was led by psychologists and medical doctors. It contained an explanation of the importance of secondary prevention in cardiovascular disease management with a duration of five minutes. Throughout this briefing, participants received guideline-coherent recommendations regarding physical activity and lifestyle modifications and the importance of smoking cessation was pointed out if the participants were active smokers^5^. Afterward, an ultrasound of the carotid arteries was conducted on each participant. Moreover, clinical parameters were assessed, containing venous blood work and measurement of the body weight and blood pressure. Finally, participants were asked to fill out a questionnaire package including a self-assessment of medication adherence. Medical history and demographic data were documented for study purposes. During each follow-up visit, the previously performed clinical examinations were repeated, including the carotid artery ultrasound to update the app representation. Changes in medical history and medication were documented. Moreover, patients were asked to re-assesses their level of physical activity. At the final follow-up visit after twelve months, an interview was conducted which contained the uMars questionnaire^28^. Using this questionnaire, the *PreventiPlaque* app was rated in order to use the results for further patient-centered app development. The results of the uMars questionnaire will be featured in an upcoming publication.

### The *PreventiPlaque* app

**Fig. 3:**
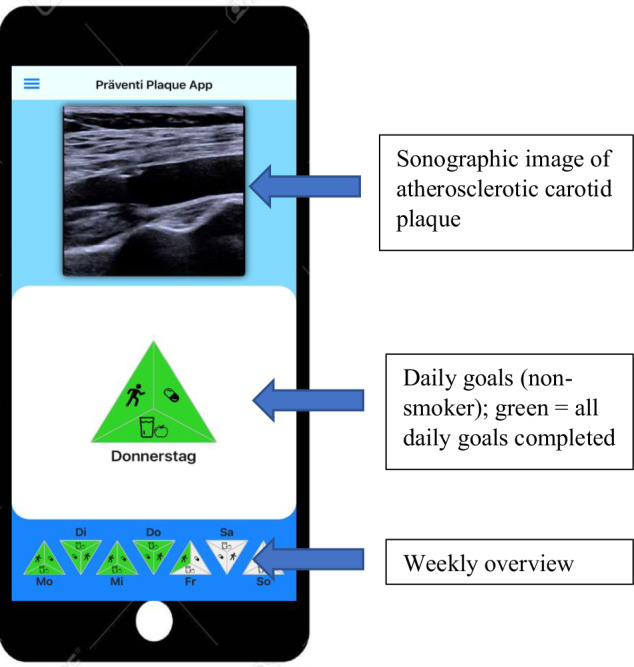
*PreventiPlaque* home screen. The displayed home screen of the *PreventiPlaque* App illustrates its core functionalities designed to support users in monitoring and reducing atherosclerotic plaque progression. The interface includes three main components: Sonographic image of atherosclerotic carotid plaque: The top section of the screen features a saved sonographic image of the user’s carotid artery, showing the presence and extent of atherosclerotic plaque. This visual serves as a personalized and educational tool for tracking vascular health. Daily Goals Section: The central triangular icon represents daily health goals, such as maintaining physical activity, adhering to a healthy diet, and avoiding smoking. A green-colored triangle indicates that all daily goals have been successfully met. This feature encourages users to maintain healthy habits consistently. Weekly Overview: The bottom row displays a compact weekly summary of the user’s goal adherence, with individual triangles corresponding to each day of the week. The color of each triangle reflects the completion status of daily goals, providing a quick visual assessment of the user’s progress over time. This overview helps reinforce accountability and long-term behavior change.

**Fig. 4:**
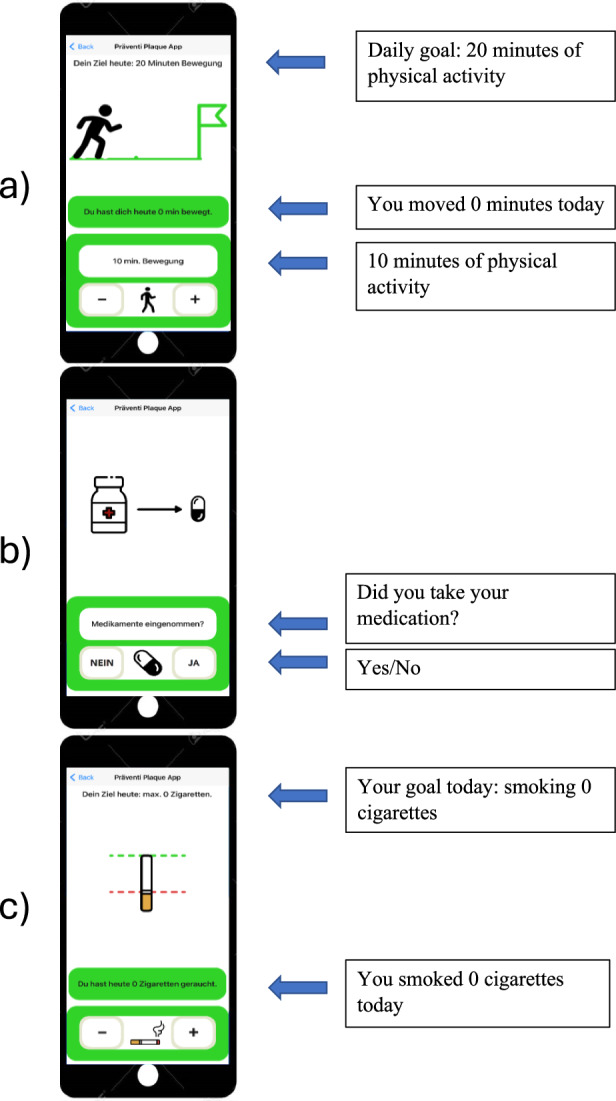
*PreventiPlaque* daily tasks. The figure illustrates three key screens of the *PreventiPlaque* App, showcasing its functionality to support daily health task management and adherence tracking for cardiovascular health improvement: **a** Daily Goal: Physical Activity (20 min): This screen highlights the user’s daily target of 20 min of physical activity. The top banner displays the goal, accompanied by a progress visualization. Below, the app shows the user’s current progress (“You have moved 0 min today”) and provides manual adjustment options using “+“ and “−“ buttons to record additional activity. **b** Daily medication reminder: This screen focuses on medication adherence. The interface includes a simple visual cue with icons for a pill bottle and a pill, accompanied by the question, “Have you taken your medication?” The user can log their adherence by selecting “Yes” or “No”. **c** Daily Smoking Goal: This screen displays the user’s smoking-related target, emphasizing abstinence with a goal of 0 cigarettes. A graphic of a cigarette and a status tracker are presented, showing progress with the message, “You smoked 0 cigarettes today.” Users can adjust their logged smoking activity using “+“ and “−“ buttons, allowing for accurate tracking.

**Fig. 5:**
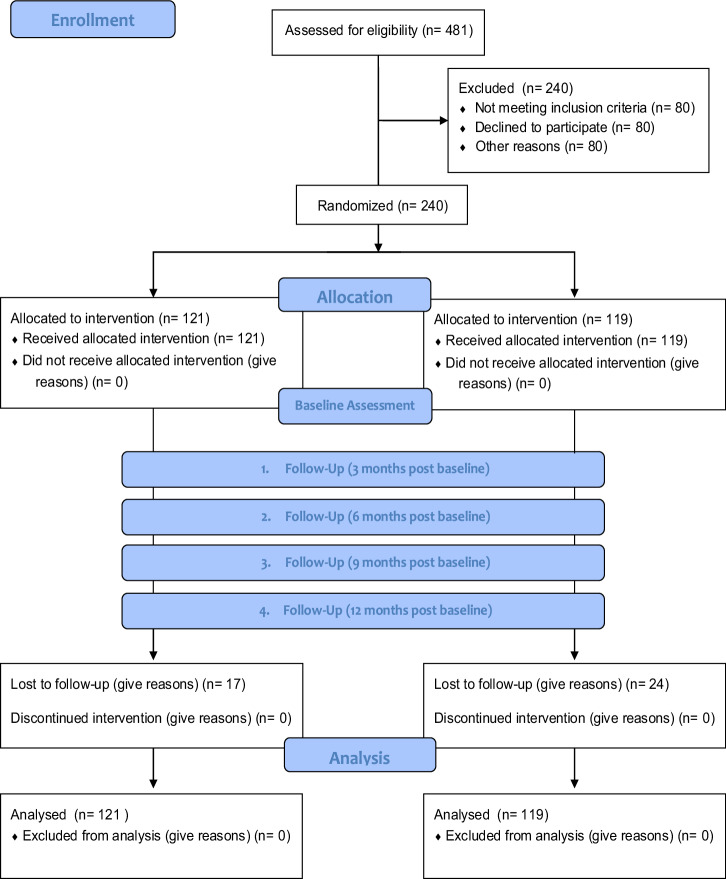
CONSORT flow chart. The CONSORT (Consolidated Standards of Reporting Trials) flow diagram outlines the progression of participants through the stages of a randomized controlled trial, including enrollment, allocation, follow-up, and analysis. A total of 480 individuals were assessed for eligibility, with 240 excluded due to not meeting inclusion criteria (*n* = 80), declining participation (*n* = 80), or other reasons (*n* = 80). The remaining 240 participants were randomized into two groups: 121 were allocated to the intervention group, and 119 to the control group, with all receiving their allocated interventions. During follow-up, 17 participants in the intervention group and 24 in the control group were lost to follow-up, with no discontinuations in either group. All participants (121 in the intervention group and 119 in the control group) were included in the final analysis, with no exclusions.

The mobile intervention *PreventiPlaque* app was solely designed for study purposes and there were no changes made to any of the included content during the study period. The only dynamic within the application was the visual presentation of the atherosclerotic carotid plaque since the ultrasound images were updated in between each follow-up visit. The primary component of the home screen was an ultrasound image of carotid atherosclerotic plaque. By clicking on the image, participants were presented with their own most-recent ultrasound images, including highlighting of the carotid plaque burden. Within the lower part, the home screen presented a weekly overview of completed low-threshold daily tasks and, by clicking, led the user to the different task subcategories (Fig. 3). Depending on smoking status, the study participants were able to record their daily progress in each of three or four health categories. The categories included physical activity, water intake, medication adherence, and—in the case of active smoking—the number of cigarettes smoked during the day. Completing all daily tasks in each health category led to a color change into green (all tasks completed), yellow (tasks partially completed), or red (no tasks completed). The health categories include the following daily subtasks:

- *Physical activity*: Participants were asked to self-record duration and type of physical activity, while choosing between activities with different intensities. Examples given were walking (light activity), cycling (moderate activity), or playing tennis (high intensity). To reach the set daily goal, 20 min of physical activity was needed (Fig. 4).
- *Drinking*: The daily water intake was supposed to be documented, which was then aligned with a previously defined water intake at the start of the study. A color change to green appeared when the target quantity was reached or missed by <10%.
- *Medication adherence*: In this category, the patients were asked to report whether they had taken the prescribed medication on the current day (Fig. 4).
- *Smoking*: This category was only displayed for participants who stated to be active smokers. In order to successfully complete this task, the participant had to smoke fewer cigarettes than the recorded number of cigarettes smoked on the previous day (Figs. 4 and 5).

### Sample size considerations and statistical analysis

To allow for loss to follow-up and missing data, estimated at 15%, a total of 240 participants were recruited. The intended power was 0.95 (post hoc *t*-test; type of power analysis: a priori; effect size *d*_RM_: 0.5; alpha error probability: 0.05; group 1 sample size: 105; group 2 sample size: 105).

Statistical analyses were performed using R (4.3.1). To examine the intention-to-treat population, missing values on primary and secondary outcome variables were imputed. The overall percentage of missing values across all outcome variables varied between 0.8% and 17.5%, with 1904 in 208,800 records (0.9%) incomplete in total. Under the assumption of missing at random, multiple imputation was used for the creation of 60 multiple imputed datasets. Incomplete outcome variables were imputed using the default settings for predictive mean matching of the ‘mice’ package^29^.

Descriptive statistics were applied for sociodemographic and medical data, as well as primary and secondary outcomes. To investigate long-term effects of the *PreventiPlaque* app on cardiovascular risk profile (SCORE2) and lifestyle alterations (LDL-C, HbA1c, systolic blood pressure, physical activity, medication adherence, quality of life), repeated measure analyses of variance (ANOVA) with a within-between factor-design (within: time, between: group) were conducted. Mauchly’s test revealed a violation of the assumption of sphericity, therefore the Greenhouse–Geisser correction (*ε*) was utilized. Post hoc tests included one-way ANOVAs for simple main effects (group, time) and *t*-tests for pairwise comparisons between single measure points within groups. *P*-values were adjusted for multiple comparisons via Bonferroni correction. Partial eta-squared (*η*^2^_p_) was used as effect size for main and interaction effects. Cohen’s *d*_Repeated Measures_ (*d*_RM_) was applied as an effect size of post hoc comparisons between measure points within groups. Effect sizes were reported and interpreted according to Cohen^30^. The level of significance was set to *α* < 0.05 for all tests.

## Data Availability

Anonymized data is available upon request.
